# Commentary: Psychological Science's Aversion to the Null

**DOI:** 10.3389/fpsyg.2017.01715

**Published:** 2017-09-27

**Authors:** Jose D. Perezgonzalez, Dolores Frías-Navarro, Juan Pascual-Llobell

**Affiliations:** ^1^Business School, Massey University, Palmerston North, New Zealand; ^2^Department of Methodology of the Behavioral Sciences, Universitat de València, Valencia, Spain

**Keywords:** data testing, hypothesis testing, null hypothesis significance testing, effect size, falsificationism, statistics

A commentary on **Psychological Science's Aversion to the Null** *by Heene, M., and Ferguson, C. J. (2017). Psychological Science under Scrutiny: Recent Challenges and Proposed Solutions, eds S. O. Lilienfeld and I. D. Waldman (Chichester: John Wiley & Sons), 34–52.*

Heene and Ferguson (2017) contributed important epistemological, ethical and didactical ideas to the debate on null hypothesis significance testing, chief among them ideas about falsificationism, statistical power, dubious statistical practices, and publication bias. Important as those contributions are, the authors do not fully resolve four confusions which we would like to clarify.

One confusion is equating the null hypothesis (H_0_) with randomness when “chance” actually resides in the sample. We can, indeed, read three different instances of randomness in the text: associated with the sample on pages 36 (trial performance) and 37; associated with the alternative hypothesis (H_A_) on page 41 (“less likely to observe mean differences…far off the true…mean difference of 0.7”); and associated with H_0_ throughout the text, starting on page 36. In reality, H_0_ simply claims a population non-effect (H_0_: Δ = 0) while H_A_ claims a constant effect (e.g., H_A_: Δ = 0.7), their corresponding distributions assuming random sampling variation in both cases. It is in the (random) sample where “chance” resides, as by chance we may pick a sample which shows a given effect (e.g., δ = 0.3) when the true effect in the population is either “0” (H_0_) or “0.7” (H_A_). Frequentist tests only assess the probability of getting the observed sample effect under H_0_ while Bayesian statistics also assesses the probability of such effect under H_A_ (e.g., Rouder et al., 2009). Therefore, the *p*-value does not inform about a hypothesis of chance but about the probability of the data under H_0_ (Fisher, 1954).

A second issue confuses power with missing true effects, something explicitly expressed on page 42 but also suggested when discussing sample sizes throughout the text (p. 36 onwards). The underlying argument is that larger sample sizes allow for achieving statistical significance so that a true effect may not be missed—something which is, at the same time, portrayed as unethical, e.g., p. 36, and ludicrous, e.g., p. 44. In reality, “we cannot manipulate population effect sizes” (p. 41), as they are deemed constant in the population (e.g., H_A_: Δ = 0.7), and a significant result at 50% power will not be missed at 80% power. As Heene and Ferguson's Figures 3.1A,C show, power simply moves the goalposts on the real line, reducing the Type II error (β), while the larger sample size also reduces the standard error. By moving the goalposts, smaller (by chance) sample effects get associated with H_A_, which is a correct association as long as there is a true population effect. Thus, power is there not to prevent missing effects due to small sample sizes but to be able to justify whether we could plausibly accept H_0_ when results are not significant (Neyman, 1955; Cohen, 1988).

A third issue is about falsificationism (pp. 35–37), which the authors argue cannot happen in psychology because we never accept H_0_, only reject it or fail to reject it. In reality, frequentist tests are logically based on *modus tollens*, the valid argument form for the falsification of statements (Perezgonzalez, 2017a). H_0_ is simply the contrapositive of our research hypothesis, and denying H_0_ allows us to affirm the latter. Therefore, frequentist tests are eminently falsificationist, attempting to disprove H_0_ via *reductio* arguments (*p*, α; Mayo, 2017). Indeed, H_0_ does not even need to be “zero” in the population: We could perfectly substitute the actual value of our H_A_, so that we may prove the theory false with a significant result (the “strong” test purported by Meehl, 1997).

A fourth issue is whether we always need to be in the position of accepting H_0_ (something argued on pages 36–37). This is not necessarily so. Just testing H_0_ as for rejecting it is suitable when we are only interested in learning about our research hypothesis (e.g., does the treatment have an effect?—Perezgonzalez, 2016). In such context, H_0_ provides a precise statistical hypothesis for carrying out the test and, because the actual parameter (Δ) is unknown, it only provides informative value via its rejection (Fisher, 1954), H_0_ acting merely as a “straw man” (Cortina and Dunlap, 1997). This testing procedure was not only developed in the context of small samples (Fisher, 1954) but the lack of a specific H_A_ precludes the control of Type II errors and of power. (A way forward would be to assess the effects warranted under H_0_—Mayo and Spanos, 2006—or to control sample size via a sensitiveness analysis—Perezgonzalez, 2017b).

If we wish to be able to accept H_0_, then we are stating that we are also interested in the potential demise of our intervention (i.e., if the treatment has no effect, we want to make sure it is akin to placebo; Perezgonzalez, 2016). This testing seems similar to Fisher's, but it requires active control of the severity with which the alternative hypothesis is to be tested (ideally, ≥80% power; Neyman, 1955; Cohen, 1988). Such control necessarily means more information—a precise alternative hypothesis (e.g., H_A_: μ_1_ – μ_2_ = 0.7, vs. H_0_: μ_1_ – μ_2_ = 0) and a specified Type II error for H_A_ (e.g., β = 0.20)—so that the power of the test can be managed (given α, β, and *N*). This approach not only allows for accepting H_0_ but also illustrates that power is only relevant for such purpose, not for rejecting H_0_. Such approach, and similar ones, have also been available since Fisher's tests of significance (e.g., Neyman and Pearson, 1928; Jeffreys, 1939).

As final note, frequentist approaches only deal with the probability of data under H_0_ [p(D|H_0_)]. If we want to say anything about the (posterior) probability of the hypotheses, then a Bayesian approach is needed in order to confirm which hypothesis is most likely given both the likelihood of the data and the prior probabilities of the hypotheses themselves (Jeffreys, 1961; Gelman et al., 2013).

## Author contributions

JDP initiated and drafted the general commentary. DF and JP contributed theoretical background and feedback. All authors approved the final version of the manuscript for submission.

### Conflict of interest statement

The authors declare that the research was conducted in the absence of any commercial or financial relationships that could be construed as a potential conflict of interest.
